# *Metapocyrtus* (*Dolichocephalocyrtus*) *aliwagwag* sp. nov., a new flightless weevil (Curculionidae, Entiminae, Pachyrhynchini) from the Aliwagwag Protected Landscape, Mindanao Island, Philippines

**DOI:** 10.3897/BDJ.13.e163127

**Published:** 2025-11-18

**Authors:** Efrhain Loidge P. Pajota, Mark John T. Pepito, Rylle G. Añuber, Graden G. Obrial, Daven Jayson D. Agbas, Jhonnel P. Villegas, Milton Norman D. Medina, Analyn A. Cabras

**Affiliations:** 1 Philippine Coleopterists Society Incorporated, TroGenILab, Davao Oriental State University, City of Mati, Philippines Philippine Coleopterists Society Incorporated, TroGenILab, Davao Oriental State University City of Mati Philippines; 2 Institute for Biodiversity and Environment, Coleoptera Research Center, University of Mindanao, Davao City, Philippines Institute for Biodiversity and Environment, Coleoptera Research Center, University of Mindanao Davao City Philippines; 3 College of Teacher Education, University of Mindanao, Davao City, Philippines College of Teacher Education, University of Mindanao Davao City Philippines; 4 College of Arts and Sciences Education, Environmental Science Department, University of Mindanao, Davao City, Philippines College of Arts and Sciences Education, Environmental Science Department, University of Mindanao Davao City Philippines; 5 Faculty of Agriculture and Life Sciences, Biology Program, Davao Oriental State University, City of Mati, Philippines Faculty of Agriculture and Life Sciences, Biology Program, Davao Oriental State University City of Mati Philippines; 6 Faculty of Computing, Data Sciences, Engineering, and Technology, Information Technology Program, Davao Oriental State University, City of Mati, Philippines Faculty of Computing, Data Sciences, Engineering, and Technology, Information Technology Program, Davao Oriental State University City of Mati Philippines; 7 Faculty of Teacher Education/ Center for Futures Thinking and Regenerative Development, Davao Oriental State University, City of Mati, Philippines Faculty of Teacher Education/ Center for Futures Thinking and Regenerative Development, Davao Oriental State University City of Mati Philippines; 8 Department of Animal Science and Food Processing, Faculty of Tropical AgriSciences, Czech University of Life Sciences Prague, Praha - Suchdol, Czech Republic Department of Animal Science and Food Processing, Faculty of Tropical AgriSciences, Czech University of Life Sciences Prague Praha - Suchdol Czech Republic; 9 Faculty of Agriculture and Life Sciences, Tropical Genomics Laboratory, Davao Oriental State University, City of Mati, Philippines Faculty of Agriculture and Life Sciences, Tropical Genomics Laboratory, Davao Oriental State University City of Mati Philippines; 10 Faculty of Agriculture and Life Sciences, Terrestrial Invertebrate Research Laboratory, University Research Complex (UResCom), Davao Oriental State University, City of Mati, Philippines Faculty of Agriculture and Life Sciences, Terrestrial Invertebrate Research Laboratory, University Research Complex (UResCom), Davao Oriental State University City of Mati Philippines

**Keywords:** Eastern Mindanao Biodiversity Corridor, Philippine endemic, Pachyrhynchini, taxonomy, mimicry complex

## Abstract

**Background:**

The genus *Metapocyrtus* Heller, 1912, is the most speciose and taxonomically complex genus in the tribe Pachyrhynchini. Currently, the genus is comprised of more than 250 species that are classified into seven subgenera. One of the least studied subgenera under the genus Metapocyrtus is the subgenus Dolichocephalocyrtus Schultze, 1925, whose members are generally known for their long and slender rostrum (0.60 to 0.76 times as long as wide). Another distinct feature of this subgenus is the evident sexual dimorphism: males possess elytra with a rounded apex and a steep apical declivity, while females exhibit a sharply pointed, triangular projection at the elytral apex. Like most weevils under the genus *Metapocyrtus*, species under the subgenus Dolichocephalocyrtus have a narrow geographic range due to their flight inability; this is probably the reason why all known species under this subgenus are endemic to the Philippines.

**New information:**

A new species of flightless weevil, belonging to the genus Metapocyrtus Heller, 1912, subgenus Dolichocephalocyrtus Schultze, 1925, from the Aliwagwag Protected Landscape (APL), Cateel, Davao Oriental, Mindanao, Philippines, is described and illustrated. Named after the Aliwagwag Falls, the novel species, Metapocyrtus (Dolichocephalocyrtus) aliwagwag Pajota & Cabras, sp. nov., is the first species of the genus to be formally described from Cateel, Davao Oriental. The description of this novel species adds to the rich diversity of the genus *Metapocyrtus* in the country and highlights the ecological significance of the Eastern Mindanao Biodiversity Corridor (EMBC).

## Introduction

*Metapocyrtus* Heller, 1912 (Heller 1912) (Coleoptera, Curculionidae) is a genus of jewel weevil belonging to the subfamily Entiminae Schönherr, 1823 (Schönherr 1823), of the tribe Pachyrhynchini Schönherr, 1826 (Schönherr 1826). The members of this genus, similar to the other members of the tribe Pachyrhynchini, are generally flightless due to the absence of their membranous and metathoracic wings. They also inhabit a very narrow and specific geographic range due to their inability to fly (Cabras et al. 2021a). The genus has its centre of distribution in the Philippines, resulting in the majority of the species being endemic to the country. Notably, a few species of this genus have also been discovered in Japan and Orchid Island, Taiwan (Cabras et al. 2022), making it a non-country-specific group. Additionally, the combined anatomical characteristics of the rostrum, pronotum and body categorise the genus into seven subgenera, including *Artapocyrtus* Heller, 1912; *Dolichocephalocyrtus* Schultze, 1925; *Metapocyrtus* Heller, 1912, s. str.; *Orthocyrtus* Heller, 1912; *Sclerocyrtus* Heller, 1912; *Sphenomorphoidea* Heller, 1912; and *Trachycyrtus* Heller, 1912 (Heller 1912, Schultze 1925, Yap and Gapud 2007, Cabras et al. 2022b).

Amongst the seven subgenera of the genus *Metapocyrtus*, the subgenus Dolichocephalocyrtus is one of the least-studied groups, as new members have only been discovered in the early years of the 21^st^ century. For 86 years, this subgenus faced taxonomic inactivity as no new species were recorded since the description of M. (D.) duyagi Schultze, 1934 (Schultze 1934). In 2020, the subgenus regained taxonomic momentum with the discovery of M. (D.) zamboanganus Cabras, Madjos & Medina, 2020, followed by five subsequent species: M. (D.) baulorum Cabras, Pajota & Medina, 2022; M.(D.) malindangensis Cabras, Pajota & Medina, 2022; M. (D.) kutongbusaw Pajota, Medina & Cabras, 2022; M. (D.) chloroglosus Rukmane–Bārbale, 2024; and M. (D.) agryoglosus Rukmane–Bārbale, 2024 (Cabras et al. 2020, Cabras et al. 2022a, Pajota et al. 2022, Rukmane-Bārbale 2024). Remarkably, four of these recently described species have been discovered in the southern part of the Philippines (Mindanao), highlighting the potential for more weevil species discoveries, primarily in the underexplored areas of the Eastern Mindanao Biodiversity Corridor (EMBC).

Mindanao is one of the principal islands and the second-largest island in the Philippines, with a land area of 94,630 km^2^. It is located in the southern part of the country and is home to a wide array of unique floral and faunal species occupying various habitat types (Pepito et al. 2020). The floral and faunal geographic and distributional range categorises the island into five sub-regions: Northeast Mindanao (Caraga Region), Central Mindanao, Western Mindanao (Zamboanga Peninsula), Southern Mindanao and Eastern Mindanao (Diesmos and Brown 2011). Several areas within these sub-regions remain entomologically underexplored, even amongst protected landscapes like the Aliwagwag Protected Landscape (APL), located in the Provinces of Davao Oriental and Davao de Oro. The APL was declared a Protected Area by Proclamation No. 139 in 2011 and is part of the Philippines’ National Integrated Protected Area System (NIPAS) and the Expanded National Integrated Protected Area System (E-NIPAS). This protected landscape blankets the upper Cateel River Basin of Mt. Agtuuganon and Mt. Pasian — a mountain complex listed as a Key Biodiversity Area (KBA) within the EMBC (Philippine Eagle Foundation et al. 2008). As a critical ecological sanctuary, the APL is open to local and foreign visitors (Mayuga 2021), offering activities such as swimming and trekking, which makes it highly susceptible to disturbance from anthropogenic activities.

In this paper, a new species of flightless weevil, belonging to the genus Metapocyrtus subgenus Dolichocephalocyrtus from Aliwagwag Protected Landscape, Cateel, Mindanao, Philippines, is described and illustrated. Notes on its habitat, intraspecific variation of scale colouration and pattern amongst individuals and a proposed mimicry complex associated with the new species are also presented.

## Materials and methods

Prior to the field expedition, a Wildlife Gratuitous Permit (No. XI-2023-31) was secured from the Department of Environment and Natural Resources (DENR) Region XI, and permission to conduct the study was granted by the Aliwagwag Protected Landscape Protected Area Management Board through Resolution No. 2, series of 2023. The specimens, currently deposited at the University of Mindanao Coleoptera Research Center (UMCRC), were collected opportunistically through bush beating and handpicking. The collected specimens were then stored in vials filled with 95% ethanol. The specimens were subsequently spread, air-dried for a few days and mounted on mounting cards with labels. Anatomical characters were observed under a Nikon SMZ745T stereomicroscope. The treatment of the genitals followed the protocol of Yoshitake (2011). Images of the habitus and genitalia were taken using a Canon EOS R10 digital camera, equipped with an MP-E 65-mm macro lens. All images were stacked using a Helicon Remote (Ver. 4.4.4 W), processed using a licensed version of Helicon Focus 6.7.0 and cleaned using Photoshop CS6 Portable software.

Label data are indicated verbatim. Measurements in this paper are abbreviated as follows:

- / = different lines;
- // = different labels;
- **ā** = arithmetic mean;
- **N** = number of specimens in the type series;
- **LB** = length of the body in dorsal view, from the anterior margin of the pronotum to the apices of the elytra;
- **LE** = length of the elytra in dorsal view, from the level of the anterior margins to the apices of the elytra;
- **LP** = length of the pronotum, from posterior to anterior margins along the mid-line;
- **LR** = length of the rostrum;
- **WE** = maximum width across the elytra;
- **WP** = maximum width across the pronotum;
- **WR** = maximum width across the rostrum.

All measurements are in millimetres (mm).

Comparative materials and specimens used in the study are deposited in the following institutions:

- **DGC** - Daven Agbas and Graden Obrial Personal Collection, City of Mati, Philippines;
- **SMTD** - Senckenberg Natural History Collections, Dresden, Germany;
- **UMCRC** - University of Mindanao Coleoptera Research Center, Davao City, Philippines.

### Geospatial Analysis and Mapping

In this study, we visualised and analysed distributional data of the new species and quantified the vegetation cover classes of Aliwagwag Protected Landscape to complement the information about the ecological status of the collection site. For the distribution map, the coordinates were gathered using the Garmin GPS Map 64x series, with a positional accuracy of ≤ 5 metres. In addition, vegetation cover was analysed using the Normalised Difference Vegetation Index (NDVI), which utilises satellite imagery with embedded spectral signature data. NDVI is a valuable index for assessing vegetation cover and its implications on the ecological status of the area. Typically, NDVI values range from -1 to +1, while, in the local study area, they vary between -0.15 and 0.53 (Khosravi et al. 2016). In this study, the satellite imagery was extracted from USGS.gov, provided by the Landsat 8 satellite with a Level 2 surface reflectance product. The downloaded data had already been atmospherically corrected to provide surface reflectance values. The satellite image was captured on 31 March 2020 and processed on 22 August 2020, using Collection 2, Tier 1 standards. This ensures that the data are of the highest quality and meet geometric and radiometric accuracy standards. To create the NDVI map, the following equation was used: NDVI = (NIR – VIS) / (NIR + VIS), where VIS and NIR represent the spectral reflectance measurements in the visible (red) and near-infrared regions, respectively (Khosravi et al. 2016).

## Taxon treatments

### Metapocyrtus (Dolichocephalocyrtus) aliwagwag

Pajota & Cabras sp. nov.

60D3425A-FACA-580C-A96A-F845E084FD42

C270CA73-BEB2-4EA5-B713-31FFA473CE99

#### Materials

- **Type status:** Holotype. **Occurrence:** recordedBy: RG Añuber; individualCount: 1; sex: Male; lifeStage: Adult; occurrenceID: 8CF9E918-DED4-5F19-8971-5587311C71E9; **Taxon:** kingdom: Animalia; phylum: Arthropoda; class: Insecta; order: Coleoptera; family: Curculionidae; genus: Metapocyrtus; subgenus: Dolichocephalocyrtus; specificEpithet: aliwagwag; **Location:** continent: Asia; island: Mindanao; country: Philippines; countryCode: PH; stateProvince: Davao Oriental; municipality: Cateel; locality: Aliwagwag Protected Landscape (APL); **Identification:** identifiedBy: ELP Pajota; AA Cabras; **Event:** samplingProtocol: Handpicking; year: 2024; month: 7; day: 17-21; **Record Level:** institutionCode: UMCRC
- **Type status:** Paratype. **Occurrence:** recordedBy: RG Añuber; individualCount: 12; sex: 4 males, 8 females; lifeStage: Adult; occurrenceID: F48700E9-A642-5AF2-8E24-D9449FE12469; **Taxon:** kingdom: Animalia; phylum: Arthropoda; class: Insecta; order: Coleoptera; family: Curculionidae; genus: Metapocyrtus; subgenus: Dolichocephalocyrtus; specificEpithet: aliwagwag; **Location:** continent: Asia; island: Mindanao; country: Philippines; countryCode: PH; stateProvince: Davao Oriental; municipality: Cateel; locality: Aliwagwag Protected Landscape (APL); **Identification:** identifiedBy: ELP Pajota; AA Cabras; **Event:** samplingProtocol: Handpicking; year: 2024; month: 7; day: 17-21; **Record Level:** institutionCode: UMCRC
- **Type status:** Paratype. **Occurrence:** recordedBy: P Camposo; S Cadayona; individualCount: 4; sex: 2 males, 2 females; lifeStage: Adult; occurrenceID: 37593EDF-415B-5130-AB85-8B5B2CDD1CD9; **Taxon:** kingdom: Animalia; phylum: Arthropoda; class: Insecta; order: Coleoptera; family: Curculionidae; genus: Metapocyrtus; subgenus: Dolichocephalocyrtus; specificEpithet: aliwagwag; **Location:** continent: Asia; island: Mindanao; country: Philippines; stateProvince: Davao Oriental; municipality: Cateel; locality: Aliwagwag Protected Landscape (APL); **Identification:** identifiedBy: ELP Pajota; AA Cabras; **Event:** samplingProtocol: Handpicking; year: 2024; month: 7; day: 17-21; **Record Level:** institutionCode: UMCRC
- **Type status:** Paratype. **Occurrence:** recordedBy: GG Obrial; individualCount: 7; sex: 4 males, 3 females; lifeStage: Adult; occurrenceID: 61CDF402-B66F-5178-B36D-2004CAE92A5A; **Taxon:** kingdom: Animalia; phylum: Arthropoda; class: Insecta; order: Coleoptera; family: Curculionidae; genus: Metapocyrtus; subgenus: Dolichocephalocyrtus; specificEpithet: aliwagwag; **Location:** continent: Asia; island: Mindanao; country: Philippines; stateProvince: Davao Oriental; municipality: Boston; locality: Barangay Simulao; **Identification:** identifiedBy: GG Obrial; DJD Agbas; AA Cabras; ELP Pajota; **Event:** samplingProtocol: Handpicking; year: 2024; month: 7; **Record Level:** institutionCode: DGC
- **Type status:** Paratype. **Occurrence:** recordedBy: DJD Agbas; individualCount: 22; sex: 15 males, 7 females; lifeStage: Adult; occurrenceID: 2827321B-3E84-5F6C-8C78-DAC3B04244B6; **Taxon:** kingdom: Animalia; phylum: Arthropoda; class: Insecta; order: Coleoptera; family: Curculionidae; genus: Metapocyrtus; subgenus: Dolichocephalocyrtus; specificEpithet: aliwagwag; **Location:** continent: Asia; island: Mindanao; country: Philippines; stateProvince: Davao Oriental; municipality: Cateel; locality: Aliwagwag Protected Landscape (APL); **Identification:** identifiedBy: GG Obrial; DJD Agbas; AA Cabras; ELP Pajota; **Event:** samplingProtocol: Handpicking; year: 2024; month: 7; day: 17-19; **Record Level:** institutionCode: DGC

#### Description

**Male. Dimensions**: LR 1.75–2.5 (Holotype: 2.0; ā: 2.06). WR 1.0–1.2 (Holotype: 1.0; ā: 1.01). LP 2.0–3.0 (Holotype: 3.0; ā: 2.79). WP 2.5–3.5 (Holotype: 3.0; ā: 3.03). LE 5.0–6.0 (Holotype: 6.0; ā: 5.5). WE 3.0–4.0 (Holotype: 3.2; ā: 3.46). N = 25 for all measurements.

**Figure 1.**
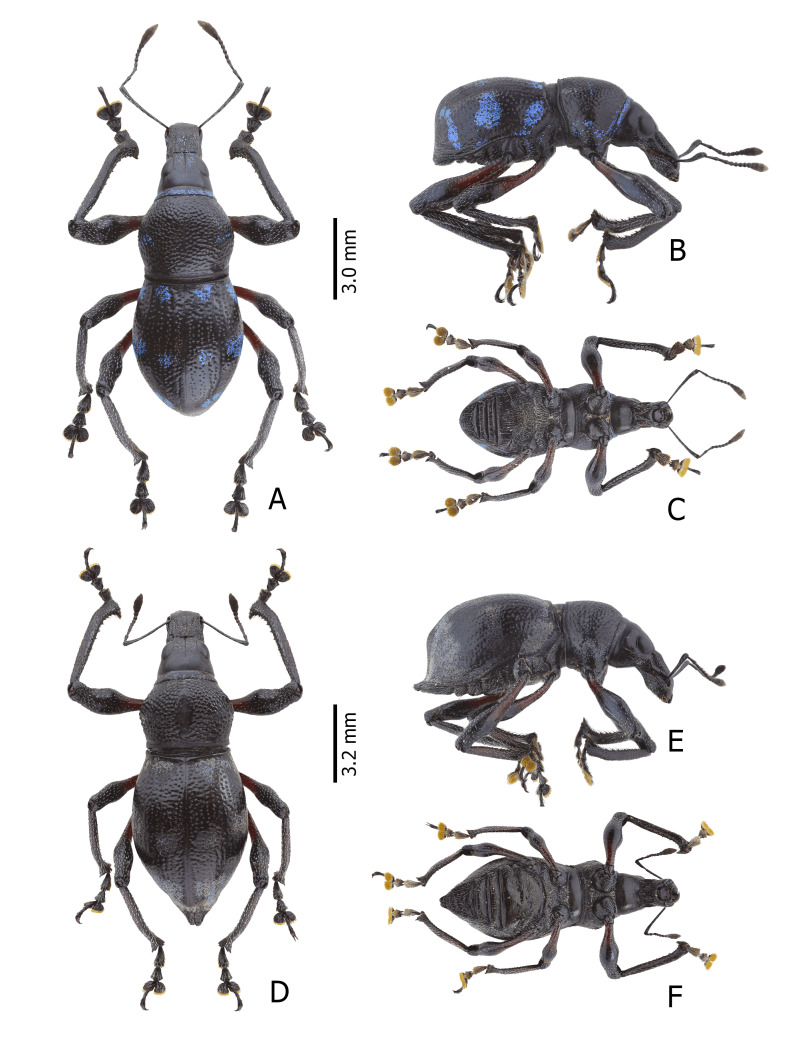
Habitus of M. (D.) aliwagwag Pajota & Cabras sp. nov.: **A** Male, dorsal view; **B** idem, lateral view; **C** idem, ventral view; **D** Female, dorsal view; **E** idem, lateral view; **F** idem, ventral view.

Habitus as shown in **Fig. 1** (A-F).

**Colouration**: Integuments are all black, except proximal half of legs, reddish-brown. Body surface, rostrum, head, legs and ventral side moderately lustrous.

**Head**: Dorsal surface finely rugose, except posterior third. Dorsal area between eyes sparsely pubescent with short sub-appressed white setae; densely beset with blue and black round and elliptical scales. Median furrow distinct, extending from apex of head to basal third, forming a vertex with transverse groove. Median furrow from posterior margin of eyes to basal third less distinct. Eyes moderately convex; area around eyes coarsely rugose. Lateroventral surface sparsely beset with short adpressed white setae and long adpressed piliform scales.

**Rostrum**: Twice as long as wide (LR/WR: 2.0/1.00); overall surface punctate – with punctures finer at apical area and coarser at basal area; basal half coarsely rugose. Dorsum surface of apical half sparsely beset with short adpressed white setae; basal half surface densely beset with long sub-erected white setae. Apicad towards mandible beset with several long erected golden setae. Median furrow present; V-shaped ridge visible at basal half – forming a shallow depression. Metallic blue round and elliptical scales present at basal half – specifically at shallow depression formed in between V-shaped ridge towards transverse groove. Hump-like protuberance formed by V-shaped ridge at base of rostrum less prominent in males than in females. Lateroventral area between oblique furrow and antennal scrobe bulging; minutely pubescent with fine sub-adpressed white setae; area below antennal scrobe densely pubescent with long sub-adpressed blue and white setae.

**Antennae**: Antennal scape slightly longer than funicle; reaching beyond posterior margin of eyes; densely beset with relatively long adpressed white setae. Funicle sparsely pubescent of long sub-adpressed white setae. Funicular antennomere 1 slightly longer than 2; approximately three times as long as wide. Funicular antennomere 3 slightly longer than three succeeding antennomeres. Funicular antennomeres 4, 5 and 6 subglobular — nearly as long as wide. Funicular antennomere 7 longer than the last four antennomeres, narrow at base and wider at apex, resembling an inverted trapezoid. Club sub-ellipsoidal; widest at middle; approximately three times longer than wide; densely pubescent with sub-erect short brown setae.

**Prothorax** subglobular; as long as wide (LP: 3.0; WP: 3.0); coarsely granulated, resembling a blackberry; mid-line groove distinct. Widest at middle, weakly convex, dorsal contour highest at posterior third. Pronotum having scaly markings of blue and black round and elliptical scales, arranged as follows: a) circular patch located at middle near dorsolateral margin, at each side of disc; b) longitudinal band of imbricate scales confluent to the band of scales at the anterior transverse groove. Dorsolateral area smooth; beset with fine white setae. Ventrolateral surface sparsely pubescent with short, adpressed white setae.

**Elytra** subovate; longer than wide (LE: 6.0; WE: 3.2); slightly wider than prothorax (WE/WP: 3.2/3.0). Elytral surface irregularly striate-punctate, with some areas sparsely pubescent with very short white setae. Dorsum widest at middle; weakly convex, with abrupt apical declivity; dorsal contour highest at middle. Declivity densely beset with much longer sub-erected white setae. Each elytron with the following scaly marking of appressed metallic blue, teal and black round and elliptical scales: a) an interrupted anterior stripe of scales forming a subcircular anterior spot near elytral suture; and a dense strip of scales diagonally placed at dorsolateral surface; b) interrupted median transverse stripe widened laterally, forming another subcircular patch of scale near elytral suture; and a dense strip of scales extending through dorsolateral surface; c) thin, interrupted transverse band at the beginning of the apical declivity, composed of clustered elliptical scales; d) a small circular patch of scales at apex, adjacent to elytral suture.

**Legs** with moderately clavate femora; basal third of femora reddish-brown, minutely pubescent with short adpressed white setae. Tibiae black, densely beset with moderately long gold and white setae; serrate along inner edges of basal third towards apex. Fore-tibiae mucronate at apex. Tarsomeres densely beset with long sub-adpressed white setae; ventrolateral area beset with longer erect brown and golden setae. Coxae beset with long sub-adpressed white setae. Pubescence in mesocoxae and metacoxae much denser than in the procoxae. Mesoventrite and metaventrite densely beset with long erected white setae; Mesoventrite prominently protruding at middle. Abdominal ventrite 1 weakly depressed towards metaventrite; densely covered with long sub-adpressed and erect fine white setae. Abdominal ventrite 3–5 sparsely covered with sub-adpressed fine white setae; abdominal ventrite 5 moderately rugo-punctate.

**Figure 2.**
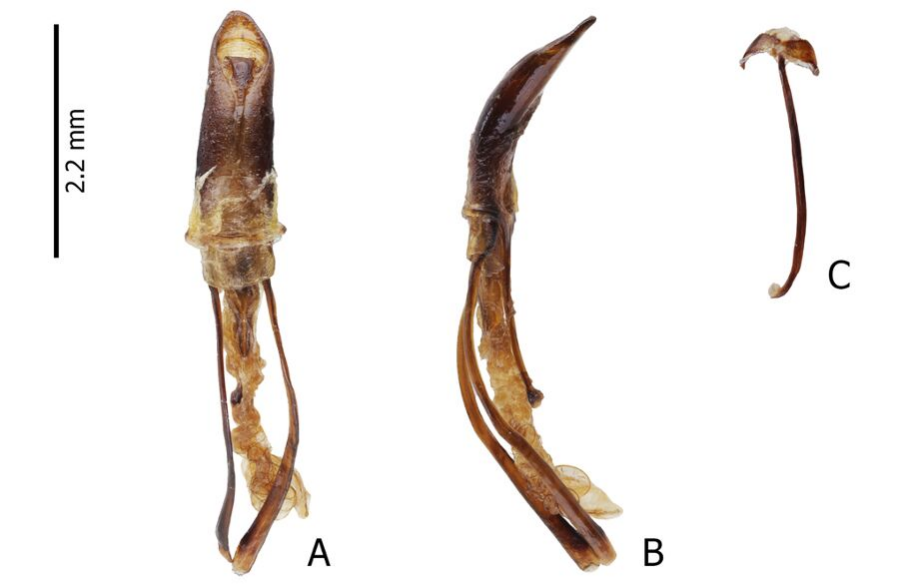
Male genitalia of M. (D.) aliwagwag Pajota & Cabras sp. nov.: **A** Aedeagus in dorsal view; **B** idem in lateral view; **C** sternite IX in dorsal view.

Male genitalia and sternite IX are shown in Fig. 2A–C.

**Female.** Dimensions: LR 1.9–2.0 (ā: 1.98). WR 1.0–1.2 (ā: 1.06). LP 2.0–3.0 (ā: 2.4). WP 3.0–4.0 (ā: 3.25). LE 6.0–7.5 (ā: 6.89). WE 3.5–5.0 (ā: 7.8). N = 20 for all measurements.

**Females** (Figs. 1D–F) differ from males, based on the following characteristics: **Rostrum** with noticeably more distinct V-shaped ridge resulting in a much deeper medial depression; hump-like protuberance at base of the rostrum significantly more prominent. Relatively wider **Prothorax**, with presence of glabrous discal area free of granulations at middle of pronotum. **Elytra** clearly wider and longer; with a distinct sutural hump-like protuberance beset with erect setae at beginning of apical declivity similar to M. (D.) kutongbusaw Pajota, Medina & Cabras, 2022; however, unlike the latter, the projected inward curve is not as concave. Furthermore, the apex of elytra bears a triangular projection, a common sexual dimorphism characteristic within the subgenus. Lastly, the second series of type specimens collected from Barangay Simulao, Boston comprises several female individuals bearing green and yellow scales, rather than the predominantly blue scales observed in the holotype and paratypes from the APL, exhibiting intraspecific variation in scale colouration within the species.

#### Diagnosis

Metapocyrtus (Dolichocephalocyrtus) aliwagwag Pajota & Cabras sp. nov. belongs to the subgenus Dolichocephalocyrtus in having a long and slender rostrum, presence of a V-shaped ridge on the basal half of the rostrum and the sharp triangular projection at the elytral apex in females, as prescribed by Schultze (1923), Yap and Gapud (2007) and Cabras et al. (2022). The new species bears a close resemblance to Metapocyrtus (Dolichocephalocyrtus) baulorum Cabras, Pajota & Medina, 2022, but can be easily distinguished from the latter, M. (D.) baulorum, by the following morphological characteristics: **A)** difference in the colour of the integument of the prothorax. M. (D.) aliwagwag Pajota & Cabras sp. nov. bears a black pronotum, while M. (D.) baulorum exhibits a reddish-brown pronotum; and **B)** the presence of short white setae inside the punctures on the surface of the elytra. In M. (D.) baulorum, these white setae are consistently present throughout the surface of the elytra, whereas in M. (D.) aliwagwag, the pubescence within the punctures is observed only on the apical declivity near the suture and along the lateral margin of the elytral apex. For the female, **C)** the new species bears a hump-like protuberance at the apical declivity, a character not exhibited by the female M. (D.) baulorum. Meanwhile, **E)** the erect white setae in the triangular projection at the elytral apex of M. (D.) aliwagwag Pajota & Cabras sp. nov. are less dense than the setation observed in M. (D.) baulorum. Moreover, the female specimen of M. (D.) aliwagwag Pajota & Cabras sp. nov. also bears a superficial resemblance to the patterns of Metapocyrtus (Metapocyrtus) subfasciatus variabilis Schultze, 1925 (Schultze 1925) from Samar Island, Eastern Visayas, Philippines, but can be easily distinguished by the hump-like protuberance at the apical declivity found in the female individuals of M. (D.) aliwagwag Pajota & Cabras sp. nov., which changes the overall shape of the elytra. Lastly, in males, compared to M. (D.) ruficollis Waterhouse, 1842 (Waterhouse 1842, Schultze 1925), M. (D.) aliwagwag Pajota & Cabras sp. nov. has a less distinct mid-line groove in the pronotum due to its coarsely granulated, blackberry-like pronotal surface, contrasting with the punctured surface of M. (D.) ruficollis Waterhouse, 1842. Another distinguishing feature between the two species is the presence of a conical projection on the ventral surface of the rostrum, which is only observed in the females of M. (D.) ruficollis and is absent in females of both M. (D.) aliwagwag sp. nov. and M. (D.) baulorum.

#### Etymology

The specific epithet *aliwagwag* is a noun in apposition, named after the Aliwagwag Falls. The Falls lie within the APL, where most type specimens, including the holotype, were collected. By naming the species after the Aliwagwag Falls, the authors underscore the ecological and conservation significance of the APL.

#### Distribution

**Figure 3.**
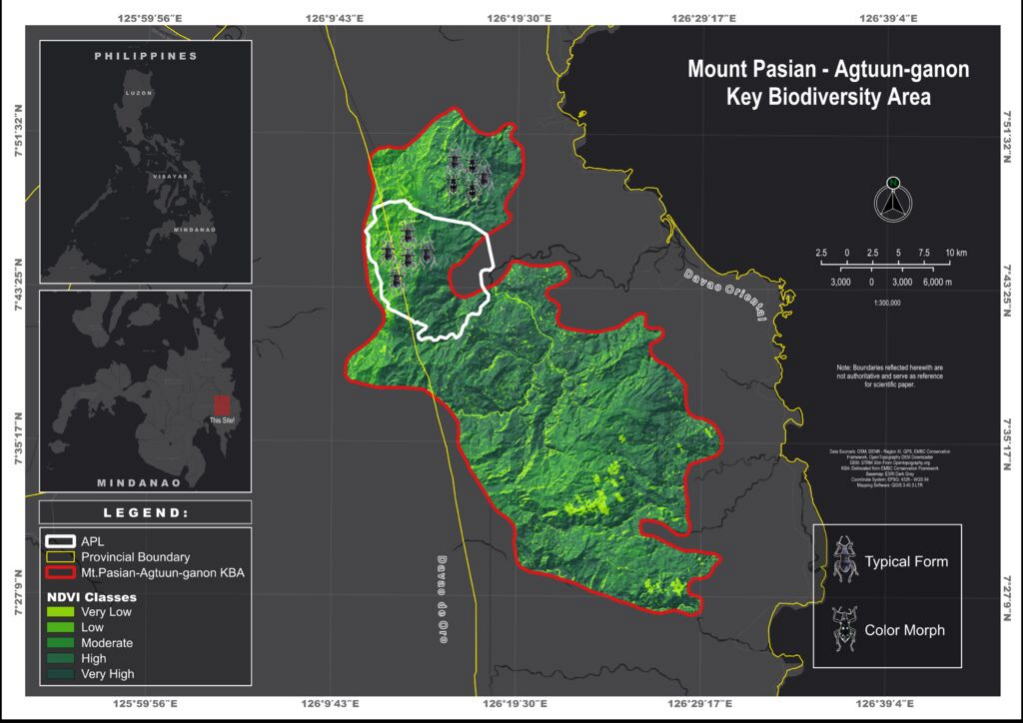
Distribution map of M. (D.) aliwagwag Pajota & Cabras sp. nov. at the Mt. Pasian – Agtuuganon Key Conservation Area, Davao Region, Philippines.

The new species described in this paper is known to occur within the Mt. Pasian-Agtuuganon Mountain complex (Fig. 3), which spans multiple districts across different municipalities in Davao Oriental and Davao de Oro. The holotype and most paratypes were collected from the APL within the territorial boundaries of Cateel, Davao Oriental, Mindanao, Philippines. Additionally, a separate series of paratypes exhibiting a yellow-coloured scale variation in females was obtained from the territorial boundaries of Barangay Simulao, Boston, Davao Oriental, Mindanao, Philippines.

#### Ecology

**Figure 4.**
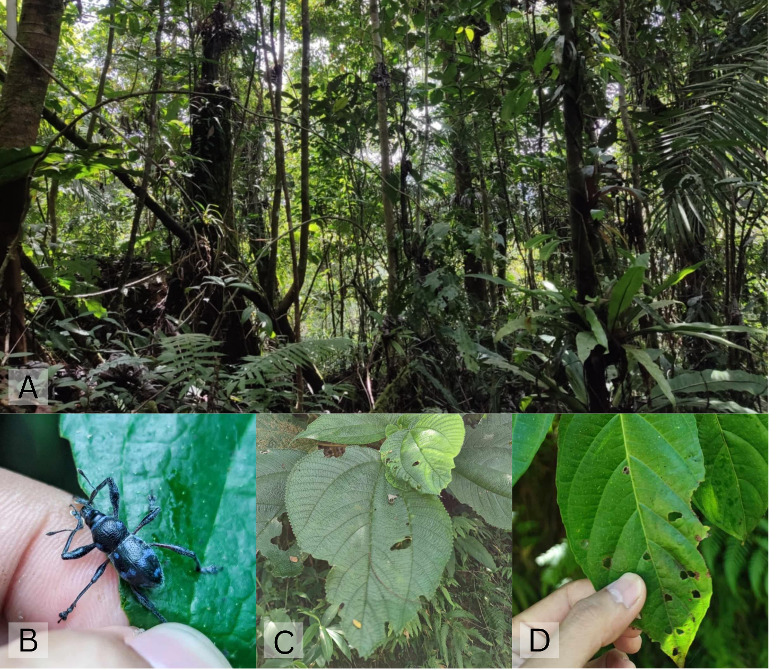
**A** Habitat of M. (D.) aliwagwag sp. nov. in the Aliwagwag Protected Landscape, Cateel, Davao Oriental, Philippines; **B-D** Species of plants associated with M. (D.) aliwagwag Pajota & Cabras sp. nov.; **B** *Chromolaena odorata*; **C**. *Cypholophus moluccanus*; **D** Ficus botryocarpa var. botryocarpa.

The type specimens of Metapocyrtus (D.) aliwagwag sp. nov. were collected from the leaves of several plants, including *Leucosyke capitellata* (Poir.) Wedd. (Urticaceae), *Ficus minahassae* (Teijsm. and de Vr.) Miq. (Moraceae), *Chromolaena odorata* (L.) R.M. King and H. Rob. (Asteraceae), *Premna cumingiana Schauer* (Lamiaceae), *Homalanthus macradenius* Pax and K. Hoffm. (Euphorbiaceae), Ficus botryocarpa Miq. var. botryocarpa (Moraceae), *Piper aduncum* L. (Piperaceae), *Ficus odorata* (Blanco) Merr.(Moraceae) and *Cypholophus moluccanus* (Urticaceae) (Fig. 4). Most of the abovementioned plant species associated with M. (D.) aliwagwag Pajota & Cabras sp. nov. are known to be pioneering native tree species, with *Chromolaena odorata*, an exotic plant species, being an exception.

Given the composition of these plant species, the ecosystem in which M. (D.) aliwagwag Pajota & Cabras sp. nov. was collected appears to be undergoing secondary succession, transitioning towards a climax stage. This process was likely triggered by the destruction caused by Typhoon Pablo in 2012, severely impacting the entire Davao Oriental Region, including the Aliwagwag Protected Landscape (APL). The species was collected at an elevation range of 600–700 m above sea level (m a.s.l.), considered moderate, but vulnerable to typhoon impacts. The collection site lies within a transition zone between the multiple-use zone and the strictly protected zone, where perennial crops and pristine forests co-exist. Regarding the proximity to water sources, M. (D.) aliwagwag Pajota & Cabras sp. nov. was found approximately 300–500 m from the nearest creek. Regarding vegetation cover, the species was collected under a canopy cover ranging from 60% to 80%, allowing sufficient sunlight penetration. This observation supports the findings of Cabras et al. (2021c), who noted that species of *Metapocyrtus* are often found along trails and ridges that are partially or fully exposed to sunlight. The sustained protection, management and conservation of the APL are critical to the hidden diversity of jewel weevils.

#### Sympatric Convergence of Patterns

Mimicry in the tribe Pachyrhynchini has been recorded as early as 1889, with Wallace followed by Schultze in the 1900s; they documented mimicry complexes involving the said tribe and other beetles from the family Cerambycidae (Wallace 1889, Schultze 1923, Schultze 1925). Apart from their rigid bodies, which act as their primary defence mechanism, Pachyrhynchine weevils, especially those in the genus *Pachyrhynchus*, have evolved stunning colouration and ornamentation on their exoskeletons. These vibrant patterns not only render them strikingly beautiful, but also serve as an aposematic signal deterring their predators, by displaying their unpalatability through their bright colouration (Tseng et al. 2014, Wang et al. 2018, Agbas et al. 2024). In Mindanao, one of the fascinating mimicries in the tribe was recorded in the papers of Cabras et al. (Cabras et al. 2018, Cabras et al. 2021b, Cabras et al. 2021c), a mimicry complex amongst the species *Doliops daugavpilsi* Barsevskis, 2014; *Pachyrhynchus tikoi* Rukmane, 2016; *Metapocyrtus pseudahirakui* Cabras & Medina, 2021; and Metapocyrtus (Orthocyrtus) hirakui Cabras, Medina & Bollino, 2021, where their elytral patterns display longitudinal lines which are observed to be mimetic.

Another recently-documented example was recorded in the paper of Obrial et al. (2024), where an interspecific mimicry complex was discovered in Mt. Candalaga, Maragusan, Davao de Oro, between the species of the genus *Metapocyrtus*, a weevil from the tribe Celeuthetini and a leaf beetle (Chrysomelidae), characterised by the brick-red and all-black colouration on their exoskeletons. On a similar note, a previous study conducted by Cabras et al. (2021c) also recorded a mimicry complex on the same mountain amongst several species of Pachyrhynchini weevils from two genera (*Metapocyrtus* and *Pachyrhynchus*), displaying similar patterns of three scaly bands on the elytra. This finding was further observed by Agbas et al. (2024), who added two new species to the said mimicry complex. In this paper, four species of the genus *Metapocyrtus*, under three different subgenera, were found to exhibit closely-similar external patterns, presumed to be mimetic in nature. These shared features are primarily defined by conspicuous circular to subcircular scaly blotches on the pronotum and elytra, as shown in Fig. 5. This proposed mimicry complex represents the first recorded instance in Davao Oriental and involves Metapocyrtus (Dolichocephalocyrtus) aliwagwag Pajota & Cabras sp. nov.; *Metapocyrtus* sp. 1; *Metapocyrtus* sp. 2 and Metapocyrtus (Sphenomorphoidea) sp. To better understand the evolutionary significance and the underlying mechanisms of this mimicry phenomenon, additional morphological and molecular studies are recommended.

#### Variation of Scales Amongst Individuals

The variation in scale colouration and even the presence or absence of scaly markings amongst individuals were notably observed amongst the specimens in the type series of M. (D.) aliwagwag Pajota & Cabras sp. nov. Most specimens collected at the APL exhibit iridescent blue scaly markings (Fig. 6a), while some individuals were observed to be completely scaleless (Fig. 6b). In contrast, the second series of type specimens collected from Barangay Simulao, Boston, displays a distinctive range of iridescent scaly markings, ranging from green to yellow-green scales (Fig. 6c).

## Supplementary Material

XML Treatment for Metapocyrtus (Dolichocephalocyrtus) aliwagwag

## Figures and Tables

**Figure 5a.**
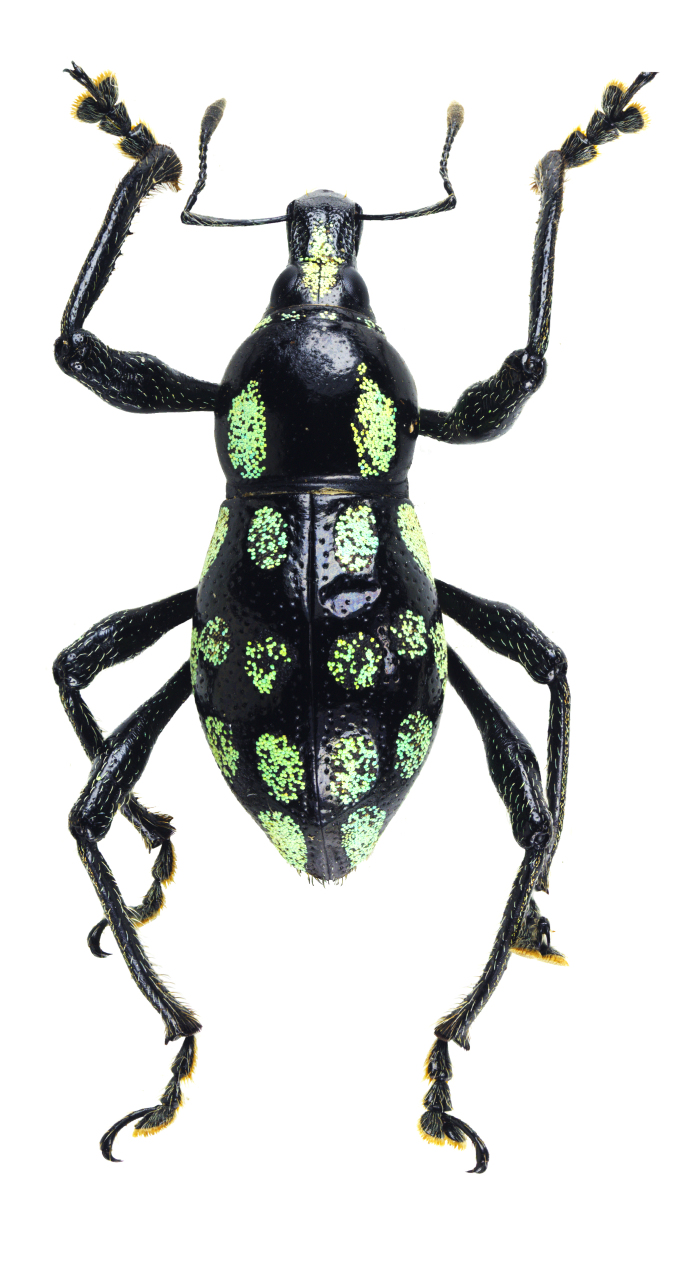
*Metapocyrtus* sp. 1;

**Figure 5b.**
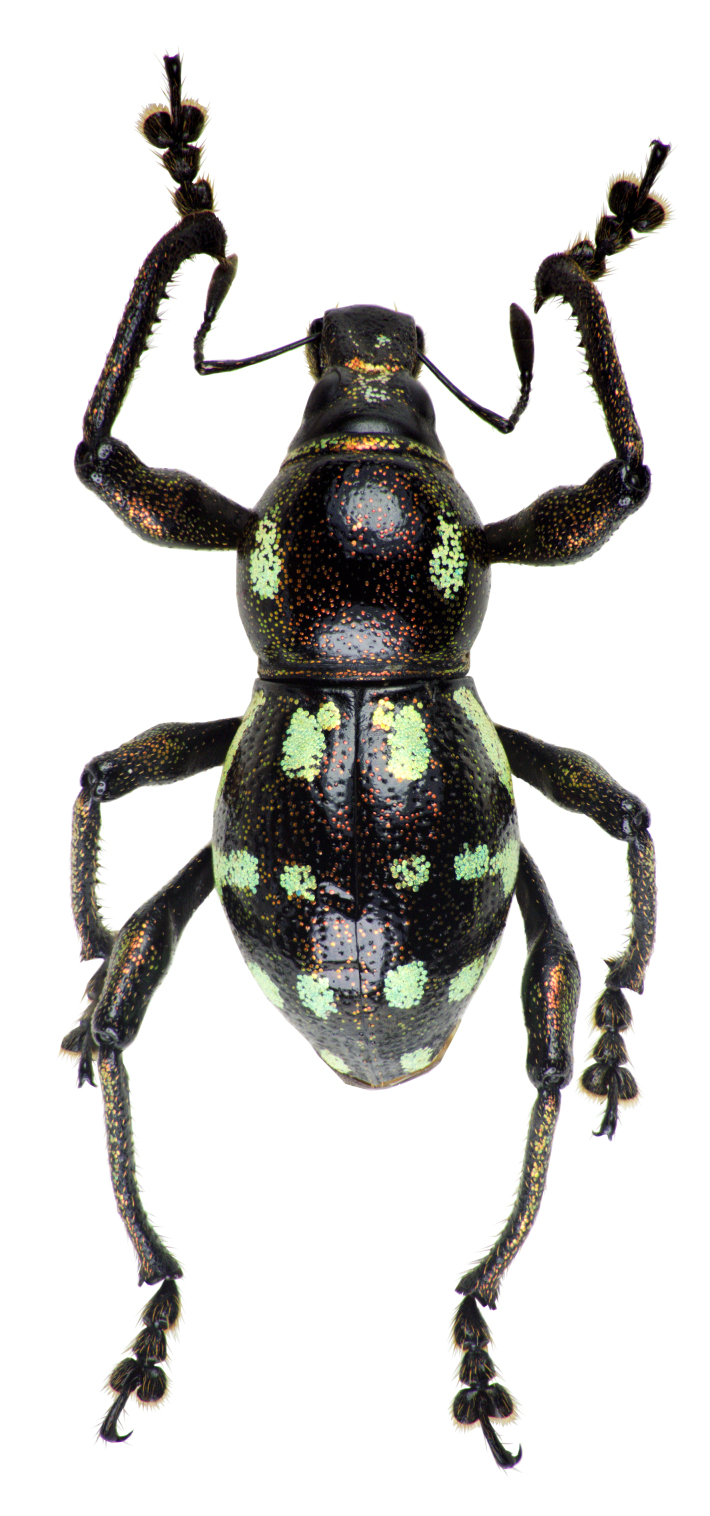
*Metapocyrtus* sp. 2;

**Figure 5c.**
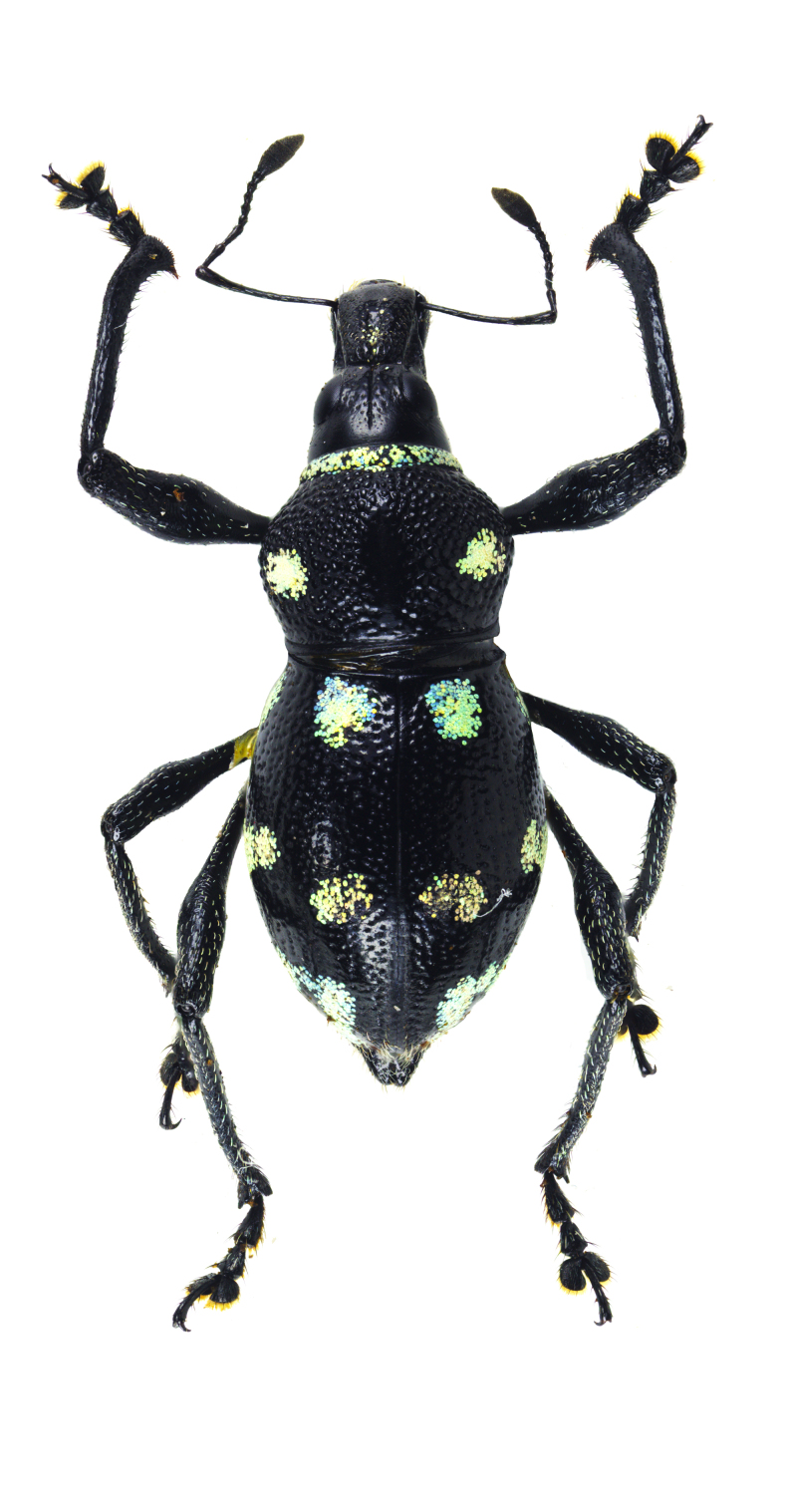
Metapocyrtus (Dolichocephalocyrtus) aliwagwag Pajota & Cabras sp. nov. (Yellow morph, collected from Brgy. Simulao, Boston);

**Figure 5d.**
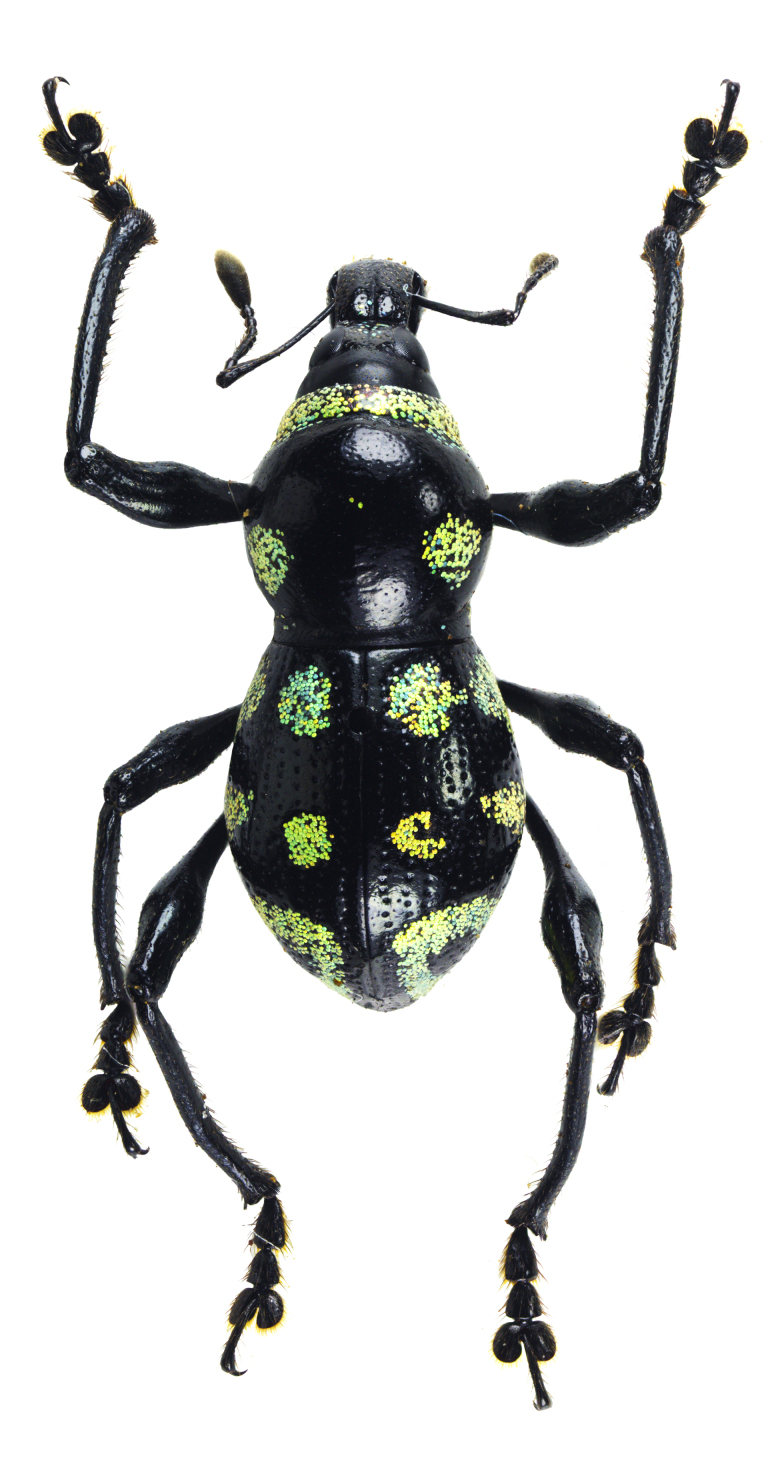
Metapocyrtus (Sphenomorphoidea) sp.

**Figure 6a.**
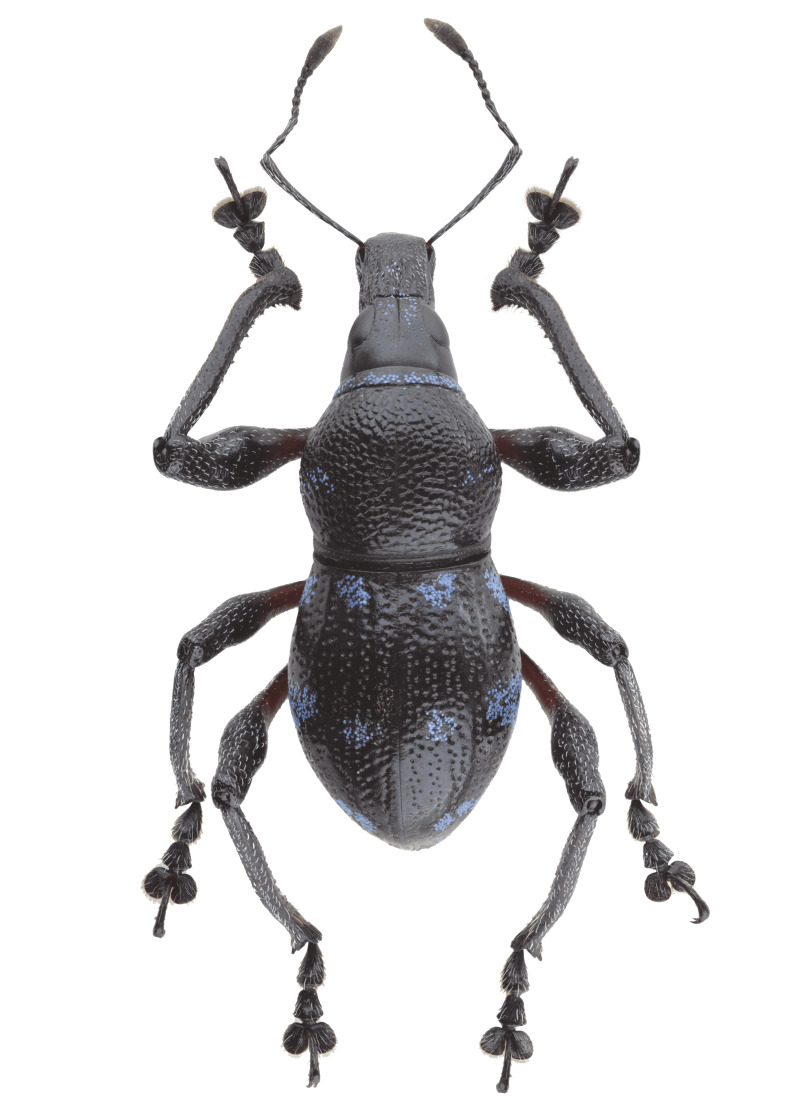
M. (D.) aliwagwag sp. nov. ♂ (Holotype);

**Figure 6b.**
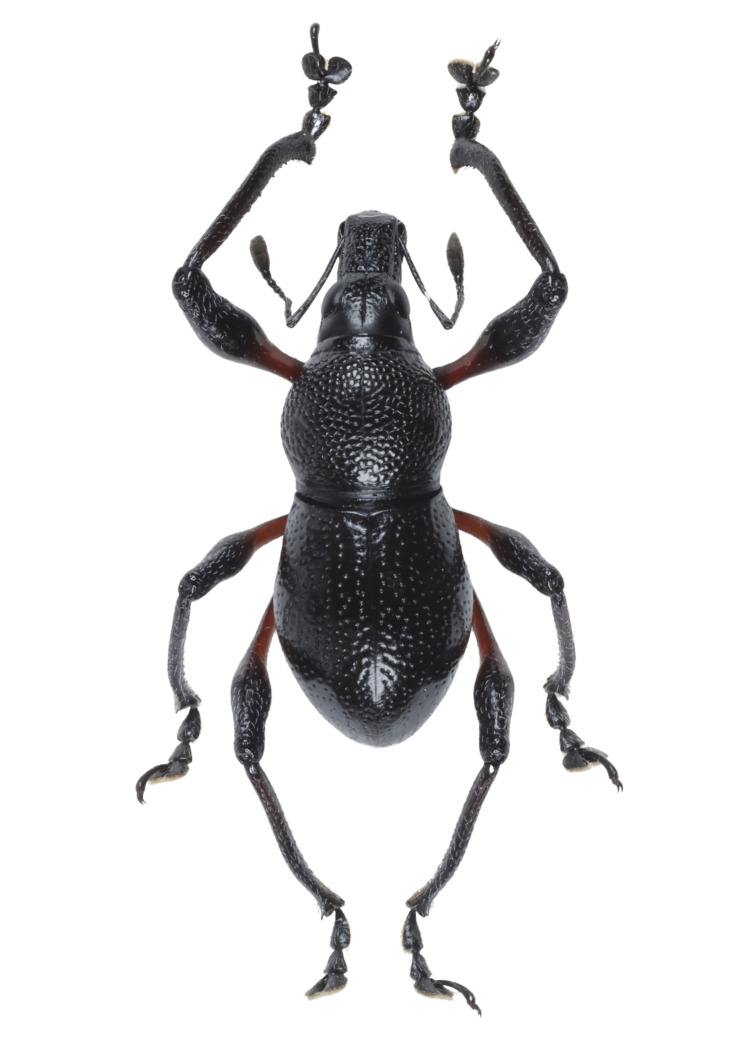
M. (D.) aliwagwag sp. nov. ♂ (Paratype; collected from the APL);

**Figure 6c.**
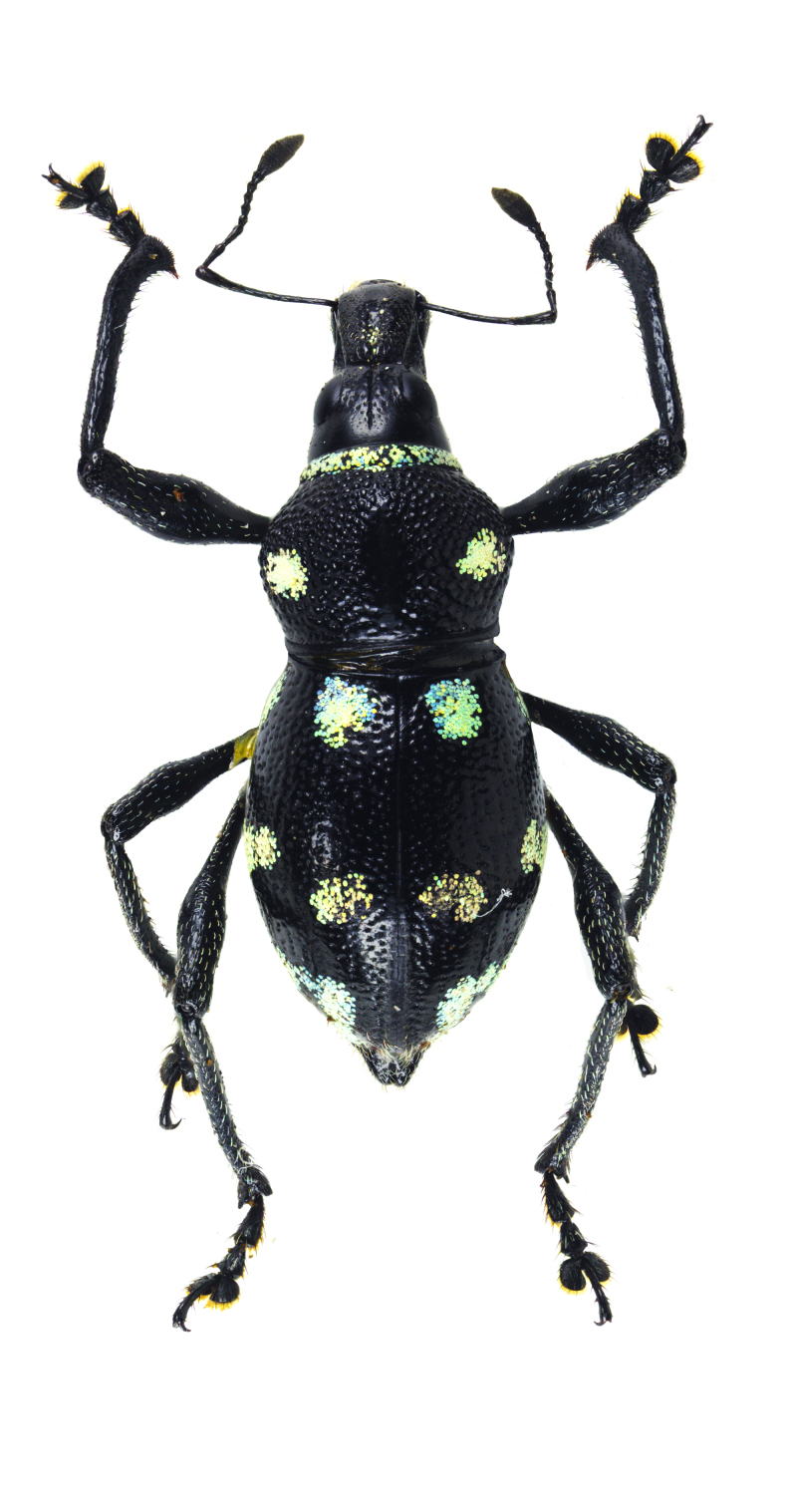
M. (D.) aliwagwag sp. nov. ♀ (Paratype; collected from Brgy. Simulao, Boston);

**Figure 6d.**
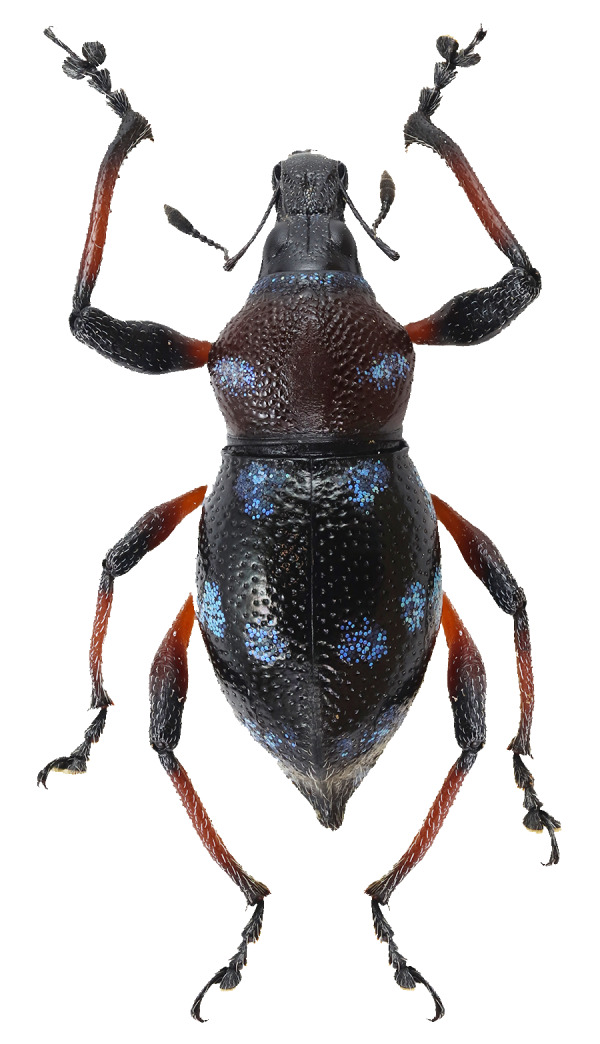
M. (D.) baulorum Cabras, Pajota and Medina, 2022 ♀;

**Figure 6e.**
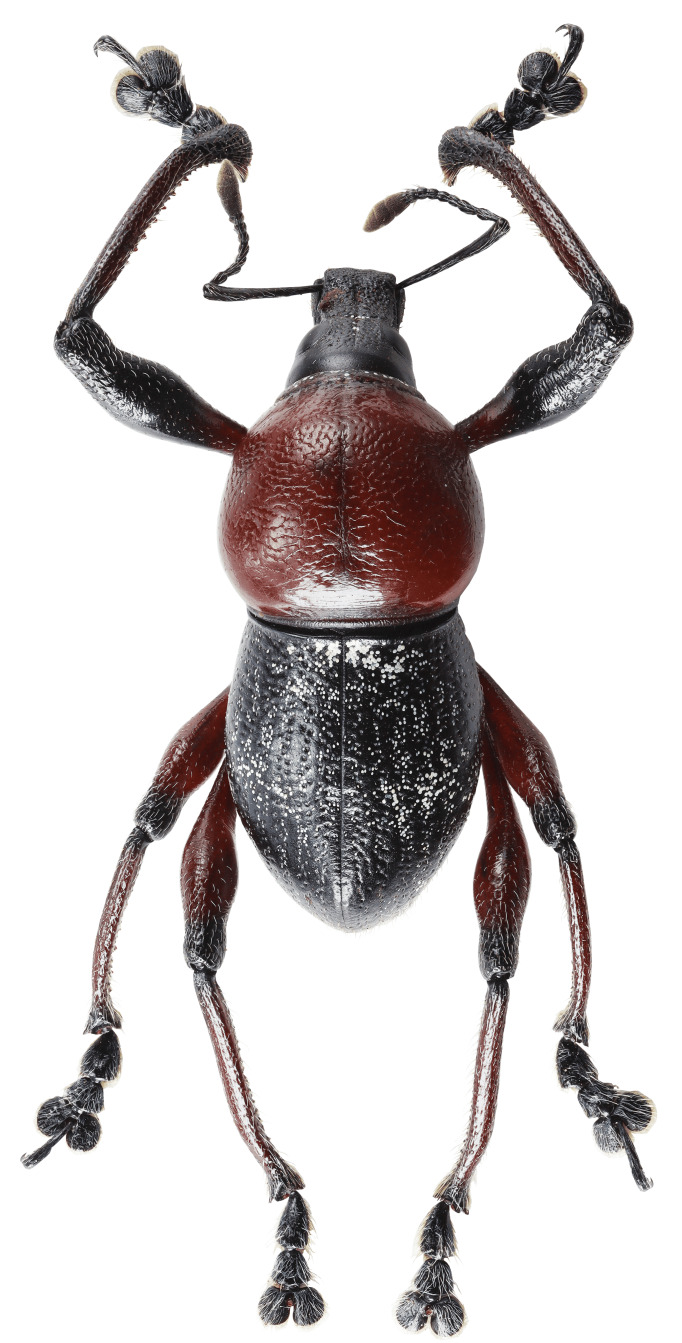
M. (D.) ruficollis Waterhouse, 1842 ♂;

**Figure 6f.**
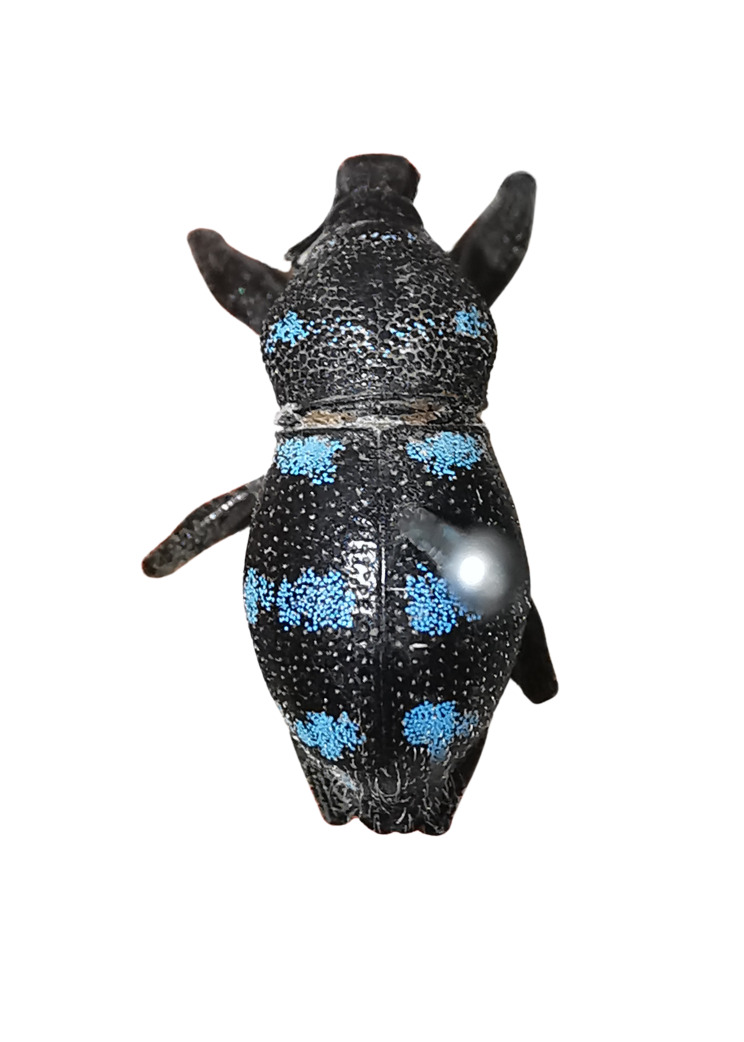
M. (M.) subfasciatus variabilis s. str. Schultze, 1925 ♀.
